# Driver’s Preview Modeling Based on Visual Characteristics through Actual Vehicle Tests

**DOI:** 10.3390/s20216237

**Published:** 2020-10-31

**Authors:** Hongyu Hu, Ming Cheng, Fei Gao, Yuhuan Sheng, Rencheng Zheng

**Affiliations:** 1 State Key Laboratory of Automotive Simulation and Control, Jilin University, Changchun 130022, China; huhongyu@jlu.edu.cn (H.H.); 17862701582@163.com (M.C.); yuhuan.sheng@hirain.com (Y.S.); 2 Key Laboratory of Mechanism Theory and Equipment Design, Ministry of Education, Tianjin University, Tianjin 300072, China; rencheng.zheng@tju.edu.cn

**Keywords:** intelligent vehicle, driver model, actual vehicle test, visual characteristics, fixation points, preview behavior

## Abstract

This paper proposes a method for obtaining driver’s fixation points and establishing a preview model based on actual vehicle tests. Firstly, eight drivers were recruited to carry out the actual vehicle test on the actual straight and curved roads. The curvature radii of test curved roads were selected to be 200, 800, and 1500 m. Subjects were required to drive at a speed of 50, 70 and 90 km/h, respectively. During the driving process, eye movement data of drivers were collected using a head-mounted eye tracker, and road front scene images and vehicle statuses were collected simultaneously. An image-world coordinate mapping model of the visual information of drivers was constructed by performing an image distortion correction and matching the images from the driving recorder. Then, fixation point data for drivers were accordingly obtained using the Identification-Deviation Threshold (I-DT) algorithm. In addition, the Jarque–Bera test was used to verify the normal distribution characteristics of these data and to fit the distribution parameters of the normal function. Furthermore, the preview points were extracted accordingly and projected into the world coordinate. At last, the preview data obtained under these conditions are fit to build general preview time probability density maps for different driving speeds and road curvatures. This study extracts the preview characteristics of drivers through actual vehicle tests, which provides a visual behavior reference for the humanized vehicle control of an intelligent vehicle.

## 1. Introduction

The driver is the main component in a driver-vehicle-road closed-loop control system. During driving, the driver obtains ~90% of environmental information through vision [1]. Specifically, the driver obtains road information by observing the external environment, makes judgments and decisions based on this information, and transforms the lane geometry information into driving operation behaviors [2]. Therefore, the visual characteristics of driving play an important role in how the driver controls vehicle movements.

Driving a car is essentially a control process; therefore, establishing a driver model based on control theory is an effective method. Current studies on intelligent vehicles typically use a driver model to simulate the vehicle control behavior of the driver and apply such models for the automatic track following of vehicles [3]. During actual driving, drivers visually perceive the area in front in the lane, judge the expected driving trajectory, and manipulate the steering wheel, accelerator, and brake pedal to follow the expected trajectory as closely as possible [4]. This is sometimes called the preview-follow process, and it is used to construct the optimal driver preview model [5]. This model simulates the driver preview behavior, controls the vehicle by extracting the information of the road ahead, and achieves a high path tracking accuracy, thereby approximating the driving behavior of drivers in reality. Visual information is a crucial input for preview control. Further, fixation information obtained by the driver serves as an important reference for the trajectory prediction of the driver control model [6]. By considering the visual behavior of the driver, the accuracy and degree of personification of the driver-preview control model can both be improved.

Studies have long investigated the eye movement behavior of drivers [7]. Eye trackers can be used to capture the fixation information of drivers through driving tests. In recent years, researchers have increasingly focused on the fixation movement law of drivers under different driving environments and vehicle movement states [8]. Many studies have found that the fixation behavior of drivers is highly correlated with the vehicle speed and road curvature [9,10]. Several researchers have suggested that driving tests conducted at extremely high speeds could clarify the fixation behavior, given that fixation becomes tightly constrained under these limiting conditions [11]. One study indicated that drivers prefer to look close to the end of the road where the road edges converge on the horizon [12]. Another indicated that the gaze distribution tends to become increasingly constrained with an increase in the driving speed [13]. This study of driver visual control used an experimental simulator to collect eye movement behavior data of drivers. However, the fidelity was greatly limited and was inadequate for fully simulating complex traffic environments, and eye movement data of drivers obtained through actual vehicle tests were lacking.

In this light, we designed an experimental scheme involving actual vehicle tests under different speeds and curvature radii. The driving tests were conducted on a straight lane and on curves with radii of 200, 800, and 1500 m, with driving at speeds of 50, 70, and 90 km/h. The selected driving conditions reflect general driving environments and provide more universal test results. We used an eye tracker to collect eye movement data of eight drivers under different driving conditions and the Identification-Deviation Threshold (I-DT) algorithm to separate the fixation points. Subsequently, to obtain more accurate fixation point coordinates under a fixed image coordinate system, we corrected and matched the eye tracker images. The image was corrected to eliminate image distortions, and the SURF algorithm was used to match the images obtained using the eye tracker and the driving recorder to reduce the impact of head movements. Furthermore, we determined the normal distribution characteristics of the fixation points and fitted the normal parameters. We accordingly extracted the preview points and projected them into the world coordinate system. Finally, we calculated the preview time probability density map under different driving conditions to provide a basis for realizing a better humanized driver control model.

The main contributions of this study are as follows:

(1) We collected eye movement data of drivers on a real road as well as the vehicle kinematics and dynamics information in real time through actual vehicle tests to overcome the insufficient reliability problem of traditional simulators. We accordingly provided a visual reference of real drivers for the humanized control of automatic driving.
(2) We experimentally tested several typical road conditions, including a straight lane and curves with radii of 200, 800, and 1500 m. We fitted the driver preview model through the preview data obtained under these conditions. We then extended the model to make it suitable for general driving conditions.
(3) From the eye movement data and image information collected using the eye tracker, we obtained more accurate fixation points through a series of image processing steps including distortion correction of the image collected by the eye tracker and matching with the image collected by the fixed position driving recorder to reduce the impact of head movements of drivers. Finally, we projected the fixation points in the image coordinate system to the world coordinate system through a bird’s-eye projection. Together, these processes enabled us to obtain more accurate fixation point coordinates, thereby improving the accuracy of the driver preview model.

The remainder of this paper is organized as follows. Section 2 discusses related works. Section 3 introduces the experimental design scheme, including the experimental equipment, choice of experimental personnel, and experimental process. Section 4 describes how the I-DT algorithm is used to separate the fixation points, distortion correction of the collected image is carried out, and fixed image coordinates of the fixation points are obtained through image matching. Section 5 analyzes the distribution characteristics of the fixation points under different working conditions, obtains the preview points and projects them to the world coordinate system, calculates the preview time, establishes the preview time distribution function, and develops the preview model. Finally, Section 6 summarizes the conclusions of this study and discusses prospects for future studies.

## 2. Related Works

A driver obtains intuitive road geometry information through their vision and converts it into driving behaviors to control the vehicle movement. Therefore, visual information is the most important source of information for drivers.

Studies have long investigated the preview behavior of drivers and fixation points. Land and Lee [14] investigated the fixation features of curve driving in a study of driving behavior and discovered specific visual cognitive characteristics of drivers. They found that drivers often look at the inner edge of the road near a so-called tangency point (TP). This is the point of geometric intersection between the inside edge of the road and the tangent passing through the position of the driver. Follow-up studies confirmed this behavior through more accurate experimental systems [15,16]. Wann and Land [17] introduced speed information into the study of fixation behavior. They visualized the optical flow from the perspective of the driver and observed so-called future points (FPs) on the expected driving path of the driver. The optical flow of FPs is always parallel to the visual direction of the driver observing the point. They reported that this is the result of conscious adjustment by the driver and noted that the driver should actually focus on the FP’s expected trajectory rather than the TP on the road edge. Lappi et al. [18] compared the possibility of driver fixation on the FP and TP based on relevant research results. They believed that both FP and TP were valid experimental fitting indexes to describe the visual cognition of the driver; nonetheless, in principle, both points could not well explain the visual cognition process of the driver. Lappi and Mole [19,20], in follow-up studies, thought that drivers did not actually fixate on a specific point and that the object of fixation information should be an area; accordingly, they developed the near–far zone theory. The far and near zones are a part of the expected trajectory before and after the TP, respectively. The driver determines the weight value of the near–far zone based on the current speed. This method combines the characteristics of the FP and the TP to provide better experimental results.

Many studies have been conducted to apply the visual characteristics of drivers to the humanized design of a better vehicle control model for an intelligent driving assistance system. Boer [21] proposed a steering control model based on the observed characteristics of driver fixation at the TP. This model uses the distance to the TP and the vehicle heading relative to the tangent vector for control. Studies on visual cognitive characteristics have provided intuitive observations of human steering behavior and further verified the rationality of the existence of fixation points and preview behavior [22,23]. Salvucci and Liu [24] conducted experimental analyses using a driving simulator to study the time process of lane changes from the steering control and eye movement behavior of the driver. Their simulation results show that after lane change starts, drivers immediately shift their main focus to the target lane and control the speed of lane change according to the shift of the fixation point. These results provide a basis for developing a new driver behavior integrated model. In a follow-up study, Salvucci and Gray designed a steering model based on the two-point visual characteristics [25]. The lane is maintained based on the near point, and the expected driving track is planned based on the far point. This steering model can adapt to the steering deviation of different drivers, has a curve recognition function, and can assist drivers in performing lateral deviation corrections and lane changes. Konstantopoulos et al. [26] recorded the eye movements of learner drivers while they drove three virtual routes that included day, night and rain routes in a driving simulator. The results show that driving instructors have more visual search strategies with a poor visual range, such as night and rainy day, which can be used to explore the visual characteristics based on driver proficiency. Wage et al. [27] analyzed glances 30 s before and 15 s after 60 naturally occurring collision warning events. They analyzed the impact of the collision warning system on the driver’s gaze characteristics when the driver is distracted or in an emergency. Sudhakar and Srinivasan [28] established a visual behavior prediction model to monitor the driver’s abnormal state such as distraction and fatigue. They also discussed some existing driver behavior models based on information on the driver’s eye movement.

The abovementioned results reveal the vast scope of the visual characteristics of drivers, and many mature analysis theories in this regard are already available. However, most studies on fixation were conducted under special environmental conditions such as typical driving conditions and special working areas; few studies investigated the fixation point distribution of drivers in general driving environments. More importantly, it is crucial to design a reasonable and effective method to obtain the fixation point while driving under all kinds of driving conditions. For most of current studies, the accuracy of the fixation information collected from some experiments could not be guaranteed owing to the lack of systematic preprocessing. Therefore, in this study, we design a method for obtaining driver preview points based on image processing through actual vehicle tests.

## 3. Actual Vehicle Experiments

### 3.1. Apparatus

**Figure 1:**
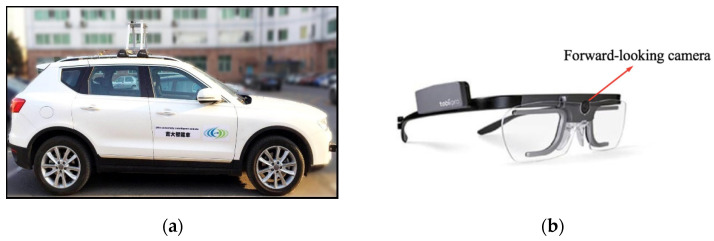
Experimental equipment: (**a**) Harvard H7 vehicle and (**b**) Tobii Glasses 2 eye tracker.

Tests were conducted using an experimental vehicle with the open controller area network (CAN) protocol. During experiments, the steering behavior information of the driver and vehicle kinematics and dynamics information were collected through a CAN acquisition card. The Tobii Glasses 2 intelligent eye tracker was used to collect viewpoints of drivers while driving. This can be used to obtain the fixation points of the driver. A forward-looking camera was installed in the middle of the eye tracker to collect images from the perspective of the driver. The eye tracker uses a noninvasive head-mounted eye tracker to ensure the comfort and freedom of the driver so as to not affect their behavior. The image collected by the eye tracker moves with the head movements of the driver. To facilitate the handling of the data, a driving recorder with a fixed viewing angle was installed under the inside rearview mirror. The stable image collected by the driving recorder was matched with the image collected by the eye tracker. In addition, the CAN bus and Kavser recorder were used to synchronously read the operation behavior of the driver and the vehicle status information. The steering wheel angle signal (rad), throttle opening (%), brake pressure (MPa), gear information, yaw rate (rad/s), and transverse and longitudinal accelerations (m/s^2^) were measured with a sampling frequency of 100 Hz. Figure 1 shows the experimental car and Tobii Glasses 2 eye tracker we used in our test.

### 3.2. Subjects

The drivers participating in this study had to meet the following requirements: (1) normal vision or habit of driving without eyewear (wearing glasses will affect the normal wearing of the head-mounted eye tracker, which may cause the collected data to be inaccurate), (2) legal driving license, (3) practical vehicle driving experience of at least 1 year, and (4) good physical and mental health. Eight drivers (six males, two females) who satisfied these requirements were finally recruited. Their average age was 27.13 years (SD = 6.70), and their average driving experience was 3.86 years (SD = 3.37).

### 3.3. Conditions

The viewpoints of the driver were collected and the visual characteristics were analyzed under different vehicle speeds and curvature radii. When passing a curve at low speed, the driving load of the driver is low and more non-driving-related eye movement behaviors may occur, which has a great impact on extracting the actual fixation points of the drivers. Considering the safety of the experiment, we did not choose the highway scene. Therefore, the test environment was conducted on urban road with safe driving speeds. Accordingly, we tested three driving speeds: 50, 70, and 90 km/h.

**Figure 2:**
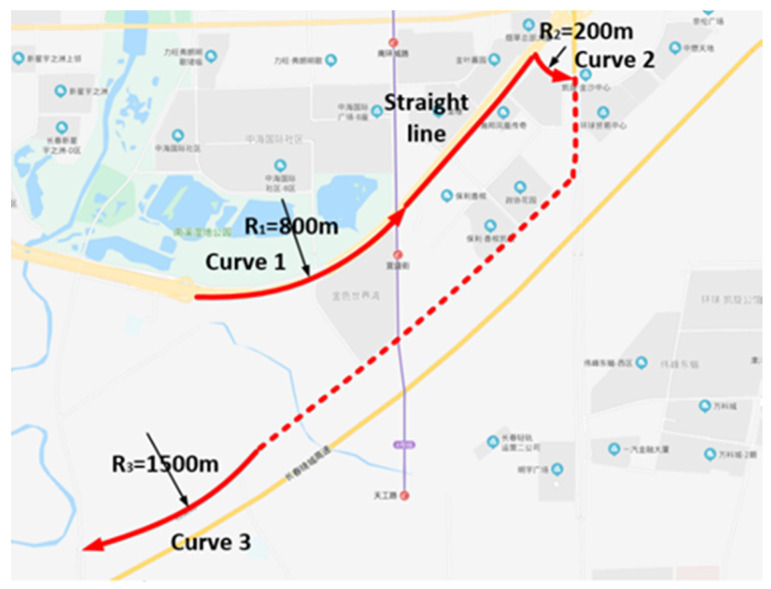
Experimental route.

The experimental conditions that had to be satisfied were as follows: road surface is properly attached, lane markings are clear, longitudinal gradient is less than 5%, lateral wind speed is less than 2 m/s, and traffic volume of selected experimental route is not too large. The route selected in the experiment should be a representative road, including straight sections, curves with small curvatures and large curvatures. The experimental route includes straight sections and curves with radii of 1500, 800, and 200 m. Table 1 lists the detailed experimental conditions. Figure 2 shows the four test sections selected in the experiment. For the test distances of the straight lane, curves with radii of 800 and 1500 m were selected for the 800-m test distance, and the curve with radii of 200 m had a test distance of 400 m. In order to ensure the continuity and authenticity of the test data, considering the sampling frequency of the eye tracker and CAN data, the test time of the driver should not be less than 10 s. Therefore, we determined the test time range shown in Table 1 according to the length of the test section and the driving speed. In addition, since we do not consider following, lane changing, overtaking, etc., it is necessary to ensure that no other vehicles interfere with the driver’s driving during the test period. For the uncertainty of traffic, driving tests needed to be repeated 2–3 times under each experimental condition. Each effective experiment must be carried out without interference from other vehicles. If there is interference from other vehicles during the experiment, we should discard this dataset and perform the experiment again.

### 3.4. Process

Before the experiments were conducted, the eight drivers drove along the experimental route to become familiar with the driving conditions and experimental instruments. Further, we used the calibration card to calibrate the eye tracker and confirm that it was working correctly. The purpose of calibration is to improve the precision of the collected data by the eye tracker. Through the calibration function of the eye tracker, the authenticity of the viewpoint information can be guaranteed to fall within a reliable accuracy range. Next, we switched on the eye movement data recording device and confirmed that it was functioning properly through the Tobii visual recording software. Subsequently, we set the basic parameters, checked the system time, connected the Kavser recorder, started the recording program, synchronized the eye movement data with the CAN data, and confirmed that the CAN data were normal.

After these preparation processes, the driver conducted the experiment by driving along the predetermined route. When approaching the test area, the driver drove the vehicle at steady speeds of 50, 70, and 90 km/h, and the experimental data were recorded.

## 4. Data Processing

**Figure 3:**
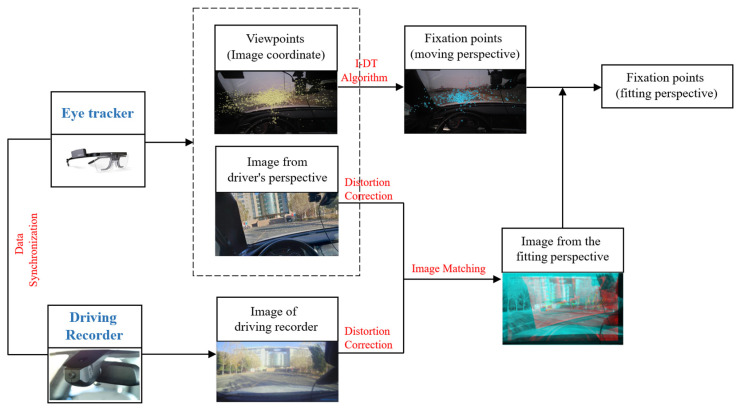
Data processing flow.

The data collected by the eye tracker include eye movement data and forward-looking images. The eye movement data include the set of viewpoints from the perspective of the driver. The desired fixation points are our focus. So, we should separate the fixation points from the viewpoint dataset first. In addition, forward-looking images were collected to obtain the locations of the fixation points in the image coordinate system. For this purpose, it is necessary to correct the distortion of the collected images. Further, because the images collected by the eye tracker move with the head movements of the driver, image matching must be performed to obtain the fixation points of the driver under a fixed perspective. Figure 3 shows the details of the data processing.

The images collected using the eye tracker and the fixed driving recorder were synchronized based on the UNIX time. Specifically, during the experiment, the UNIX timestamp of each frame of data was recorded for each group of acquired data. After synchronization, the overall synchronization error can be less than 1 ms.

### 4.1. Fixation Point Acquisition

Previous studies found that people switch their gaze from one object to another through a process called viewpoint switching [29]. When switching between the two viewpoints, our eyes are relatively stationary for ~0.2 s at a fixation point. Further, for switching from one viewpoint to the next, the eye moves rapidly, and the input sensitivity of visual information is reduced; therefore, visual information generally cannot be obtained effectively at this time. The viewpoints in the scanning process are scan points.

The original viewpoints collected by the eye tracker include fixation points and scan points. Among them, the scan points record the viewpoint switching process of drivers. Scan points are more often caused by movements of the line of sight. For understanding the driving road, we focus more on the fixation information of the driver. Therefore, it is necessary to separate the fixation points from the original viewpoints. Among the various separation methods that can be used for this purpose, the I-DT algorithm has fewer parameters, a low application difficulty, and high calculation speed [30]. Further, it can usually even be calculated in real time and has a relatively high accuracy. This study uses the I-DT method to obtain the actual fixation points of drivers.

The I-DT algorithm classifies fixation points and scan points by considering the fact that fixation points have low eye movement speed and tend to be closely clustered. The main principle used to judge a fixation point is to calculate the deviation in a moving window. The deviation includes the horizontal and vertical dispersion of the viewpoints—that is, the sum of the horizontal and vertical distances between the viewpoints and the window. If it is lower than the deviation threshold, the viewpoints in the time window are considered fixation points. The deviation is calculated as

(1) D=(max(x)−min(x))+(max(y)−min(y))

where *max*(*x*) and *max*(*y*) are, respectively, the maximum values of the *x* and *y* coordinates of the viewpoints in the window and *min*(*x*) and *min*(*y*) are, respectively, the minimum values of the *x* and *y* coordinates of the viewpoint in the window.

**Figure 4:**
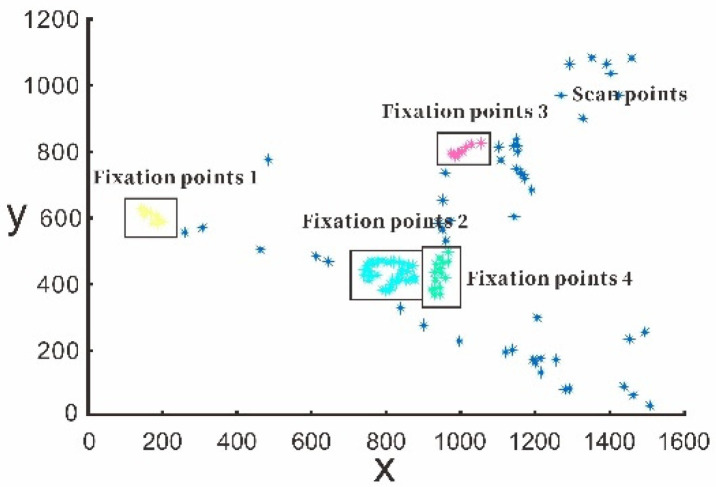
Fixation point acquisition using Identification-Deviation Threshold (I-DT) algorithm.

Figure 4 shows the separation method of the I-DT algorithm. First, the time window is initialized, the initial value is set to 0.2 s, and the viewpoint deviation threshold is set to 50. The viewpoint deviation in the window is calculated using Equation (1). If it is lower than the threshold, the viewpoints in the time window are considered fixation points. Then the time window is updated, data for the next time point are included in the dynamic time window dataset, and the next threshold calculation is performed. If the threshold value is higher than or equal to the deviation threshold, the time window is moved. The time window starts from the last time T + 1 of the previous window, and the window size is 0.2 s. The next threshold calculation is then performed. In Figure 4, during the test time of 3 s, the driver performed four fixation behaviors based on the I-DT algorithms.

**Figure 5:**
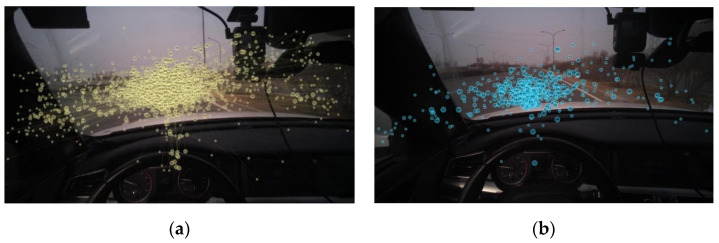
Separation results of I-DT algorithm: (**a**) original viewpoints and (**b**) separated fixation points.

Figure 5 shows the results of the separation of fixation points using the I-DT algorithm in our actual vehicle experiments.

### 4.2. Image Distortion Correction

The original image collected from the camera has a certain degree of distortion. The farther away from the image center, the greater the distortion. Image distortion adversely affects the subsequent projection transformation from the image coordinate system to the world coordinate system. Therefore, image distortion must be corrected in the data preprocessing stage [31]. The distortion model described by the image coordinate system is generally defined as

(2) $$\begin{array}{ccc} x_{u} & = & x_{c}+\frac{x_{d}-x_{c}}{T\left(r_{d}\right)}\\ y_{u} & = & y_{c}+\frac{y_{d}-y_{c}}{T\left(r_{d}\right)} \end{array}$$

where ($x_{u}$,$\;y_{u}$) is the pixel coordinate of the ideal image, ($x_{d}$,$\;y_{d}$) is the pixel coordinate of the corresponding distorted image, ($x_{c}$,$\;y_{c}$) is the coordinate of the distortion center,$\;T\left(r_{d}\right)$ is the distortion function, and $r_{d}=\sqrt{{\left(x_{u}-x_{c}\right)}^{2}+{\left(y_{u}-y_{c}\right)}^{2}}$ is the distance between the pixel and the distortion center.

The general form of the distortion function is

(3) $$T\left(r_{d}\right)=1+k_{1}r_{d}^{2}+k_{2}r_{d}^{4}+k_{3}r_{d}^{6}+\cdots L$$

Many studies have shown that an image coordinate accuracy of 0.1 pixel can be achieved by retaining only the term of the distortion parameter $k_{1}$, which is sufficient to describe the image distortion degree in the experiment [32]. Therefore, Equation (3) can be simplified as

(4) $$T(r_{d})=1+k_{1}r_{d}^{2}$$

As long as the distortion parameter $k_{1}$ is determined, the image distortion correction can be performed to obtain the corrected image coordinate. $k_{1}$ can be estimated from the third-order polynomial of $r_{d}$ in the image coordinate system [32]:

(5) $$k_{1}=\frac{r_{u}-r_{d}}{r_{d}^{3}}$$

where $r_{u}=\sqrt{x_{u}^{2}+y_{u}^{2}}$ is the center radius.

**Figure 6:**
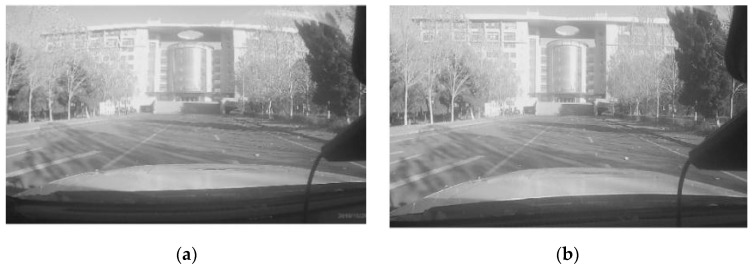
Image distortion correction: (**a**) original image and (**b**) image with distortion correction.

The corrected image can be obtained by performing pixel-by-pixel transformations using Equation (2). Figure 6 shows a comparison of the camera before and after correction. After eliminating the distortion, the camera can more accurately reflect the original appearance of the object and obtain more accurate results in the subsequent transformation to the world coordinate.

### 4.3. Image Matching

To obtain the world coordinates of the fixation points, it is necessary to determine the spatial position of the camera. However, when the driver wears the eye tracker, this position inevitably moves during driving. Further, different drivers have different heights and sitting postures. The image obtained by the eye tracker is directly transformed by projection and the camera height, pitch, roll, and other parameters are not fixed; therefore, the eye tracker may collect dynamic images.

Previous studies used a manual point-by-point verification method to eliminate the impact of movement. However, this approach required considerable time to process experimental data, making it difficult to increase the amount of experimental data and resulting in an overly large accidental error. In this study, a driving recorder with a fixed view angle was installed under the inside rearview mirror to synchronously collect image data and match the image collected by the eye tracker to eliminate this adverse effect.

The SURF algorithm was used to match images from different perspectives [33]. The main advantages of the SURF algorithm are its high matching speed and high precision. The SURF algorithm uses a Hessian matrix to calculate the eigenvalue of each pixel and identifies the stable edge points of the image using a discriminant. These edge points are actually those where the local maximum value is obtained by the discriminant of the Hessian matrix.

First, the Hessian matrix is constructed for each pixel:

(6) $$\begin{array}{l} Hes=\left[\begin{array}{cc} L_{xx} & L_{xy}\\ L_{xy} & L_{yy} \end{array}\right]\\ \det(Hes)=L_{xx}L_{yy}-L_{xy}L_{xy} \end{array}$$

The function L=f(x,y) is the gray value of a pixel (x,y). According to the discriminant det(Hes) of the Hessian matrix, the pixels satisfying the maximum value are selected, and the selected maximum point is determined as the feature point for SURF feature extraction. After finding the feature point, the SURF algorithm describes it by using a unique feature vector that does not change with the perspective transformation so as to facilitate the subsequent matching. The solution of the eigenvector is obtained as described below.

First, the main direction value is found. With the feature point as the center, a fan-shaped window with an opening angle of 60° is set, the sliding window is rotated with a step length of ~0.2 rad, and the sum of wavelet features is counted. The direction with the maximum sum of wavelet features is considered the main direction. The feature sum is found by accumulating the Harr wavelet response values dx and dy of the image to obtain a vector $m_{w}$:

(7) $$m_{w}=\sum_{w}dx+\sum_{w}dy$$

(8) $$\theta_{w}=\arctan(\sum_{w}dx/\sum_{w}dy)$$

The main direction is the direction corresponding to the maximum Harr response cumulative value—that is, the direction corresponding to the longest vector, and it is defined as

(9) $$\theta =\theta_{w}|\max\left\{m_{w}\right\}$$

Next, the eigenvector value is calculated. A square box is selected around the feature point; its diagonal direction is the main direction of the feature point. It is divided into 16 regions. In each region, the horizontal and vertical Haar wavelet characteristics of 25 pixels are counted, all of which are determined relative to the main direction of the square box. The Haar wavelet response of the image must be calculated to generate feature point descriptors. The Haar wavelet response is calculated in a rectangular region. Taking the feature points as the center, the image is divided into 4 × 4 sub-blocks along the main direction. Each sub-block uses the Haar template to calculate the response value. The feature vectors corresponding to the sub-blocks are as follows:

(10) $$V_{sub-block}=\left[\sum dx,\sum \left|dx\right|,\sum dy,\sum \left|dy\right|\right]$$

Finally, we need to find a perspective transformation to match as many feature points and feature vectors as possible. The RANSAC algorithm is commonly used for this purpose [34]; it assumes that the data comprise normal points and outliers. Outliers refer to data that are unsuitable for the hypothesis model, and they do not have any impact on the results. The specific implementation methods are as follows:

1. Four feature points are randomly selected as initial values, and a homography matrix is calculated using these points; the calculation result is unique:
  (11) $$\left[\begin{array}{c} x_{10}\\ y_{10}\\ 1 \end{array}\right]=H\left[\begin{array}{c} x_{20}\\ y_{20}\\ 1 \end{array}\right]$$
  where $\left(x_{20},y_{20}\right)$ is the coordinate of the initial feature point selected in the image to be matched, $\left(x_{10},y_{10}\right)$ is the coordinate of the initial feature point selected on the reference image, and **H** represents the homography matrix.
2. All other feature points are transformed by the homography matrix:
  (12) $$\left[\begin{array}{c} x_{1}^{\prime }\\ y_{1}^{\prime }\\ 1 \end{array}\right]=H\left[\begin{array}{c} x_{2}\\ y_{2}\\ 1 \end{array}\right]$$
  where $\left(x_{2},y_{2}\right)$ is the coordinate of the feature point in the image coordinate system of the viewing angle of the driver, and $\left(x_{1}^{\prime },y_{1}^{\prime }\right)$ represents the result of the $\left(x_{2},y_{2}\right)$ projection transformation using the homography matrix.
3. Error analysis is performed on the feature matching points of the corresponding reference image $\left(x_{1},y_{1}\right)$ of $\left(x_{1}^{\prime },y_{1}^{\prime }\right)$, and the homography matrix is output if the following formula is satisfied. Here, the error *S* is sufficiently small, and *n* is the total number of feature points:
  (13) $$S=\frac{\sum \sqrt{{\left(x_{1}^{\prime }-x_{1}\right)}^{2}+{\left(y_{1}^{\prime }-y_{1}\right)}^{2}}}{n}\leq 10$$
4. If the error analysis result *S* of this matching feature point is greater than that of the previous one, the calculation result at this time is discarded directly. Otherwise, the homography matrix is retained and the next iterative calculation is performed until the least error analysis result *S*, that is, the homography matrix with the highest matching degree, is selected as the transformation matrix.
5. According to the obtained homography transformation matrix, the fixation points in the image coordinate system under the moving perspective can be transformed into the modified fixation point coordinates in the fixed image coordinate system:
  (14) $$\left[\begin{array}{c} x^{\prime }\\ y^{\prime }\\ 1 \end{array}\right]=\left[\begin{array}{ccc} h_{11} & h_{12} & h_{13}\\ h_{21} & h_{22} & h_{23}\\ h_{31} & h_{32} & h_{33} \end{array}\right]\left[\begin{array}{c} x\\ y\\ 1 \end{array}\right]$$
  where $\left[\begin{array}{ccc} h_{11} & h_{12} & h_{13}\\ h_{21} & h_{22} & h_{23}\\ h_{31} & h_{32} & h_{33} \end{array}\right]$ is the transformation matrix, (x,y) is the fixation point coordinate in the image coordinate system under the moving perspective, and $\left(x^{\prime },\;y^{\prime }\right)$ is the modified fixation point coordinate in the fixed image coordinate system after matching.

## 5. Preview Modeling

### 5.1. Invalid Data Elimination

Because fixation point acquisition is based on actual vehicle tests, some errors inevitably occur due to the second task or distraction of drivers while driving, which could affect the experimental results. The adverse impact of these errors on the statistical analysis must be eliminated.

**Figure 7:**
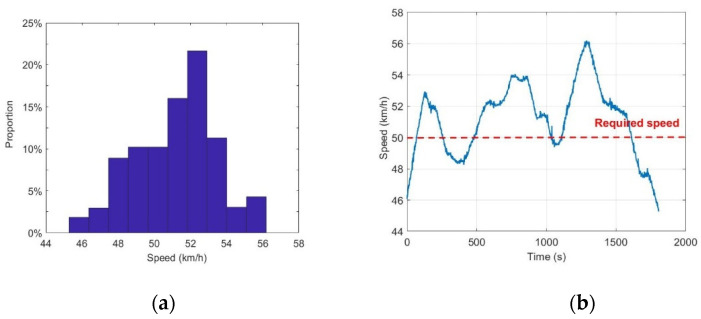
(**a**) The distribution of speed and (**b**) the speed curve of subject 1 on a straight lane; the required speed is 50 km/h.

During the experiment, the driver drives at a fixed speed as far as possible; however, to ensure the regularity and accuracy of the fixation points, the driver is not required to pay attention to the instrument panel to ensure that the speed is fixed. Therefore, for each group of data, the speed is allowed to fluctuate within a certain error range. Specifically, if the vehicle speed error of 95% of the sampling points in a given group of experimental data is less than ±10%, this group can be considered valid for further analysis of the eye movement information; if the error exceeds 10%, this group is considered invalid and is discarded. In the test of subject 1 on a straight lane with a speed of 50 km/h as an example, we recorded his driving speed throughout the experiment. Figure 7 shows the speed distribution during the experiment. According to the image analysis, the reorganization speed data are distributed within the allowable range of error, which meets the speed requirements, so the reorganization data are effective. By using this method, the error of velocity distribution of eight subjects was verified one by one. The results show that the speed distributions of all subjects are within the allowable range.

**Figure 8:**
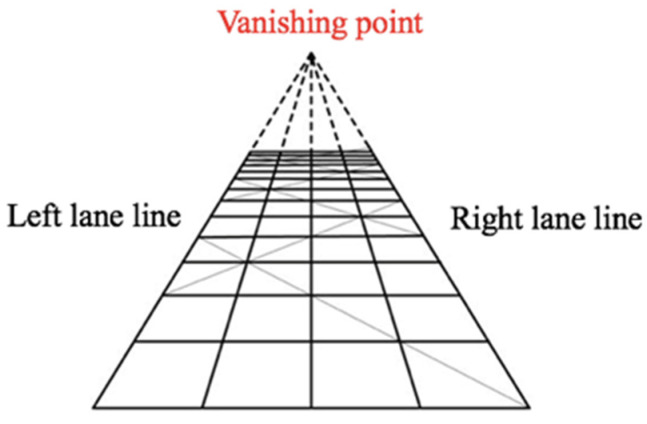
Vanishing point.

In addition, the fixation points during driving should be distributed below the vanishing point, defined as the intersection point of the left and the right lane lines in the image acquired by the camera and the end point of the road in the field of vision, as shown in Figure 8. When the calculated vertical height of the corrected fixation points is less than the vanishing point height and these points are located in the current lane, the preview of the driver falls on the driving road (i.e., the driver has reasonable preview behavior); fixation points higher than the vanishing point are discarded as invalid data.

### 5.2. Fixation Point Distribution

**Figure 9:**
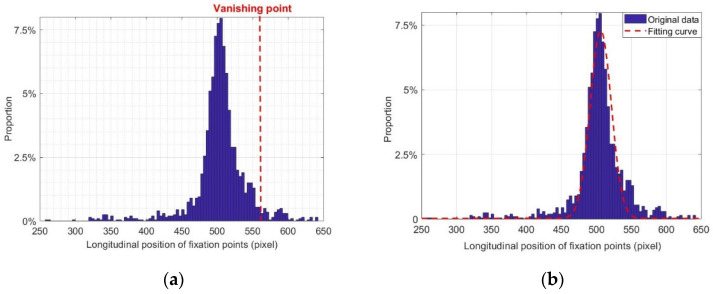
Fixation point distribution of subject 1 on a straight lane at 50 km/h: (**a**) vanishing point test and (**b**) fitting of distribution curve.

We took the experimental data of subject 1 in a straight lane as an example to study the fixation point distribution. By quantifying the longitudinal distribution of fixation points and taking 10 pixels as the statistical interval, we obtained the statistical distribution map of the fixation point of subject 1 at a speed of 50 km/h on a straight lane, which is close to the normal distribution shown in Figure 9. In order to obtain a more objective evaluation, the Jarque–Bera method, which is based on the skewness and kurtosis of data, was used to test the normality of the fixation point distribution. The Jarque–Bera test is a test for the goodness of fit of the sample data with the skewness and kurtosis consistent with the normal distribution. The statistical test results are always non-negative. If the result is much greater than zero, it means that the data do not have a normal distribution. In statistics, a normal statistic with a critical value of 0.05 is generally used as the test standard. Jarque–Bera statistics are calculated as follows [35]:

(15) $$JB=\frac{N}{6}(W^{2}+\frac{{\left(K-3\right)}^{2}}{4})$$

where the skewness is W=μ3μ232 and kurtosis is K=μ4μ22.

For ∀j>1, the J-order central moment is $\mu_{j}=\frac{1}{N}\underset{i}{\sum }{\left(x_{i}-\mu_{0}\right)}^{j}$. The first-order central moment or sample mean is $\mu_{0}=\frac{1}{N}\underset{i}{\sum }x_{i}$. Further, N is the total number of samples and $x_{i}$ is the sample to be tested. The Jarque–Bera statistics follow the $\chi^{2}$ distribution.

The Jarque–Bera statistic of the fixation point distribution of subject 1 is 1.9981, which is lower than the critical value at a significance level of 0.05. The fixation point distribution is considered to follow a normal distribution.

The Jarque–Bera method was similarly applied for tests on a straight lane at speeds of 50, 70, and 90 km/h to test the normality of the fixation point distributions of all subjects. Table 2 shows the test results. It can be seen from Table 2 that the Jarque–Bera statistic of subject 8 at a speed of 50 km/h is greater than the critical value of 5.7268 at the significance level of 0.05, so this group of data were discarded.

After proving that the fixation points have a normal distribution, the longitudinal position $y^{\prime }$ can be fitted to obtain the mean and standard deviation of the distribution:

(16) $$u=\frac{1}{\sqrt{2\pi }\cdot w}e^{-2\cdot {\left(\frac{y^{\prime }-y_{0}^{\prime }}{w}\right)}^{2}}$$

where u is the probability density of the corresponding independent variable $y^{\prime }$, $y^{\prime }$ is the longitudinal coordinate of the fixation points of drivers, w is the standard deviation of the longitudinal distribution of the fixation points, and $y_{0}^{\prime }$ is the ordinate of the distribution center of fixation points, both of which are parameters to be fitted. The spatial position of $y_{0}^{\prime }$ is the center point of the whole fixation area; therefore, this point is the preview point of the driver while driving.

### 5.3. Parameter Fitting for Straight Lane

**Figure 10:**
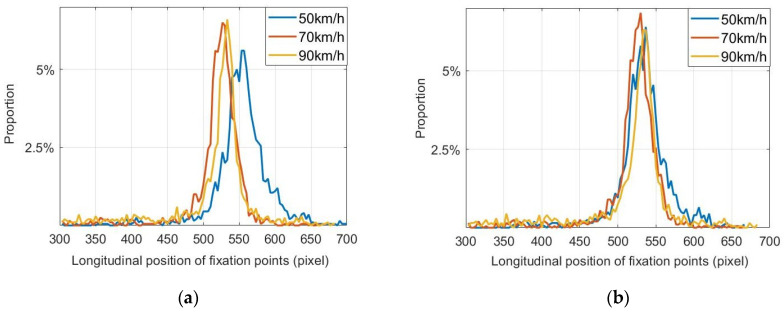
Fixation point distributions of (**a**) subject 1 and (**b**) subject 2 on a straight lane.

The fitting data of the fixation point distributions of different drivers on a straight lane were calculated. Figure 10 shows the statistical distributions of the fixation points of subject 1 and subject 2 at different speeds on a straight lane. The statistical distributions of the other six subjects also conformed to a similar normal distribution. The parameter values w and $y_{0}^{\prime }$ of each normal function can be fitted from the statistical distribution chart. w is the standard deviation of the longitudinal distribution of fixation points, and $y_{0}^{\prime }$ is the ordinate of the distribution center of fixation points.

**Figure 11:**
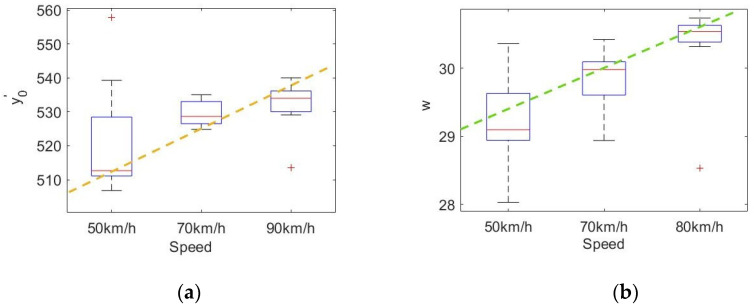
Relationship between fitting parameters and vehicle speed on straight lane: (**a**) relationship between velocity and $y_{0}^{\prime }$ and (**b**) relationship between velocity and w.

Figure 11 shows the relationship between the fitting parameters and the different vehicle speeds for different drivers. $y_{0}^{\prime }$ was fitted at each speed. It can be seen from Figure 11 that the relationship between $y_{0}^{\prime }$ and the speed *v* is approximately linear. We can further judge the linear relationship by calculating the correlation coefficient between them. The fitted straight line we performed has been showed in the figure. The results indicate that the fitting coefficients are 435.06 and 0.625. The specific relationship was

(17) $$y_{0}^{\prime }=435.06+0.625\times v$$

where the parameters 435.06 and 0.625 are fixed coefficients in the fitted straight line.

$y_{0}^{\prime }$ is the longitudinal position of the center point of the longitudinal coordinate distribution of the fixation points. Because the fixation points have a normal distribution, it is also the average value of the longitudinal coordinates of the fixation points of the driver. With an increase in the vehicle speed, $y_{0}^{\prime }$ also increases. When the speed increases, the position of the preview point becomes distant. Except for abnormal data, most viewpoints of the driver have this feature.

The difference in w at each speed was obvious. The correlation coefficient was 0.7383 (*p* ≤ 0.05), indicating a significant linear correlation. The specific relationship was

(18) w=25.24+0.105×v

where the parameter 25.24 and 0.105 are fixed coefficients in the fitted straight line.

u is the standard deviation of the longitudinal coordinate distribution of the fixation points. With an increase in the vehicle speed, w also increases, indicating that the dispersion of the fixation points increases with the speed. The distribution range of the fixation points of the driver in the longitudinal direction can be linearly fitted as a function of the speed correlation as follows:

(19) $$\begin{array}{ccc} u & = & \frac{1}{\sqrt{2\pi }\cdot w\cdot e^{-2{\left(\frac{y^{\prime }-y_{0}^{\prime }}{w}\right)}^{2}}}\\ w & = & 25.24+0.105\times v\\ y_{0}^{\prime } & = & 435.06+0.625\times v \end{array}$$

### 5.4. Parameter Fitting for Curves

The statistical and analytical methods for the fixation point distribution on a curve are the same as those for a straight lane. The fixation points of drivers are mostly concentrated near the TP of the curve. The larger the curvature radius, the more obvious this phenomenon becomes. To accurately understand the distribution characteristics of viewpoints on a curve, we need to further quantify and statistically analyze the fixation point distribution.

**Figure 12:**
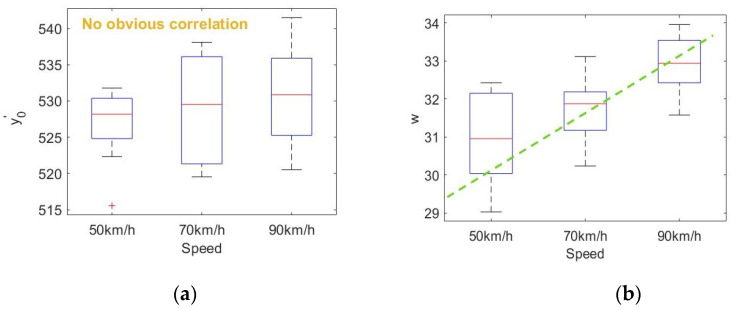
Relationship between fitting parameters and vehicle speed for curve with *R* = 800 m: (**a**) relationship between velocity and $y_{0}^{\prime }$ and (**b**) relationship between velocity and w.

First, the influence of speed on the fitting data of the fixation point distribution is determined. Taking a curvature radius of 800 m as an example, the fixation point distribution parameters under different speeds are fitted. Figure 12 shows the relationship between the fitting parameters of the fixation point distribution and the vehicle speed for a curve with *R* = 800 m. No significant difference was seen in $y_{0}^{\prime }$ at each speed, and the correlation coefficient with the vehicle speed was 0.2499, which does not indicate an obvious correlation. The difference in w at each speed was obvious and showed an obvious linear correlation with the vehicle speed. The correlation coefficient was 0.6472 (*p* ≤ 0.05), and the specific relationship was

(20) w=28.46+0.049×v

where the parameters 28.46 and 0.049 are fixed coefficients in the fitted straight line.

w is the standard deviation of the longitudinal coordinate distribution of fixation points. With an increase in the vehicle speed, w increases, the dispersion of fixation points increases, and the driver expands the fixation range. However, the speed has no significant effect on the fixation center.

**Figure 13:**
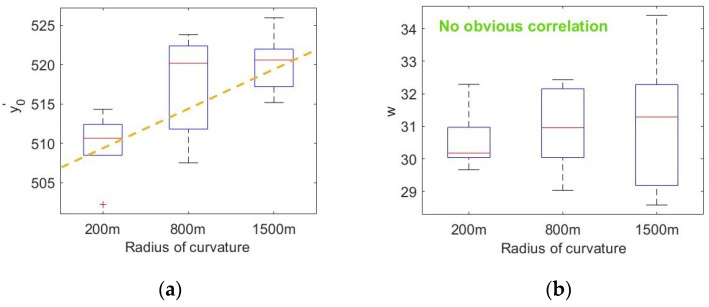
Relationship between fitting parameters and radius of curvature: (**a**) relationship between radius of curvature and $y_{0}^{\prime }$ and (**b**) relationship between velocity and w.

Next, the influence of the curvature radius on fitting parameters is analyzed. Figure 13 shows the relationship between the fitting parameters of the curve distribution and vehicle speed for different drivers at the same speed and different curvature radii. The difference in $y_{0}^{\prime }$ was obvious at each speed. The correlation coefficient between $y_{0}^{\prime }$ and radius of curvature *R* was 0.5939 (*p* ≤ 0.05) after removing the abnormal fitting data of the curve with *R* = 800 m. Relevant studies have found that the logarithm of the radius of curvature can better reflect the road curvature directly perceived by drivers [29]. The logarithm of curvature radius will not change the relationship between the properties and parameters of the curve radius, and it can eliminate the heteroscedasticity through logarithmic transformation and increase the correlation between variables, which can better reflect the driver’s visual statistical law in curve driving. The correlation coefficient between $y_{0}^{\prime }$ and the logarithm of the radius of curvature was 0.7210 (*p* ≤ 0.05), indicating a significant linear correlation, and the specific relationship was

(21) $$y_{0}^{\prime }=482.26+5.32\times \ln(R)$$

where the parameters 482.26 and 5.32 are fixed coefficients in the fitted straight line.

No significant difference was seen in w at each speed. The correlation coefficient between w and the radius of curvature was 0.1283. According to Equations (16), (20), and (21), the distribution function of fixation points on the curve is

(22) $$\begin{array}{ccc} u & = & \frac{1}{\sqrt{2\pi }\cdot w\cdot e^{-2{\left(\frac{y^{\prime }-y_{0}^{\prime }}{w}\right)}^{2}}}\\ w & = & 28.46+0.049\times v\\ y_{0}^{\prime } & = & 482.26+5.32\times \ln(R) \end{array}$$

### 5.5. Coordinate Transformation of Preview Point

In the previous study, we obtained the fixation range through the experimental data, counted the distribution characteristics of fixation points, and determined the center of the fixation area—namely, the preview point. However, these data are from the perspective of the driver—that is, the data in the image coordinate system. To establish a preview model, it is necessary to use a bird’s-eye projection to transform the coordinates of the preview point and project from the image coordinate system to the world coordinate system.

To simplify the calculation, we ignore the inclination of the road surface and use a two-dimensional coordinate to represent the preview position of the driver on the road, which is the spatial position of the preview point we want to obtain. As in the case of image matching, considering that the transformation from the perspective image of the driver to the two-dimensional road surface is also a perspective transformation, we only need to solve the perspective matrix $H^{\prime }$ by using the spatial position, focal length, and other parameters of the camera and express it in the general form of the homography matrix:

(23) $$H^{\prime }=\left[\begin{array}{cccc} f & 0 & x_{0} & 0\\ 0 & f & y_{0} & 0\\ 0 & 0 & 1 & 0 \end{array}\right]\left[\begin{array}{cc} R & T\\ 0 & 1 \end{array}\right]=\left[\begin{array}{cccc} h_{11}^{\prime } & h_{12}^{\prime } & h_{13}^{\prime } & h_{14}^{\prime }\\ h_{21}^{\prime } & h_{22}^{\prime } & h_{23}^{\prime } & h_{24}^{\prime }\\ h_{31}^{\prime } & h_{32}^{\prime } & h_{33}^{\prime } & h_{34}^{\prime } \end{array}\right]$$

where $\left(x_{0},y_{0}\right)$ is the center coordinate of the image, *f* is the focal length of the camera, and **R** is the orthogonal rotation matrix of the camera position, which can be expressed as

(24) $$R=\left[\begin{array}{ccc} r_{11} & r_{12} & r_{13}\\ r_{21} & r_{22} & r_{23}\\ r_{31} & r_{32} & r_{33} \end{array}\right]$$

**T** is the translation matrix of the camera position, which can be expressed as

(25) $$T=\left[\begin{array}{c} t_{x}\\ t_{y}\\ t_{z} \end{array}\right]$$

**Figure 14:**
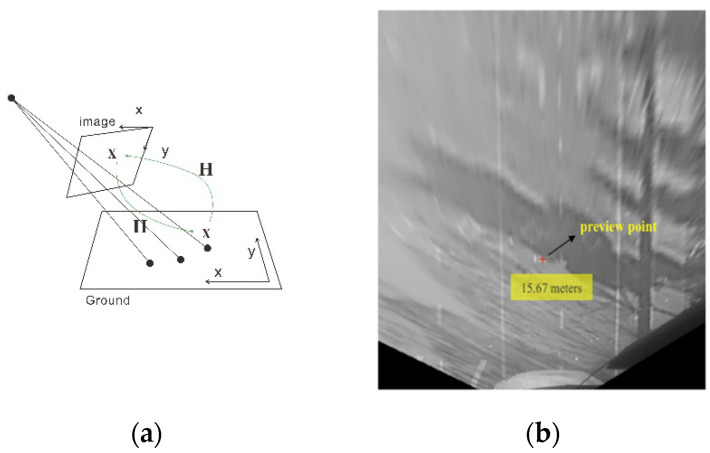
(**a**) Schematic diagram of projection transformation. (**b**) Preview point after projection.

Accordingly, the projection of the preview point in the fixed image coordinate system can be transformed to the world coordinate system as follows:

(26) $$\left[\begin{array}{c} x_{g0}\\ y_{g0}\\ 1 \end{array}\right]=H^{\prime }\left[\begin{array}{c} x_{0}^{\prime }\\ y_{0}^{\prime }\\ 0\\ 1 \end{array}\right]$$

where $\left(x_{0}^{\prime },y_{0}^{\prime }\right)$ is the preview point in the image coordinate system and $\left(x_{g0},y_{g0}\right)$ is the fixation point projected into the world coordinate system. Figure 14 shows a schematic diagram of the bird’s-eye projection and a preview point projected by this method. In Figure 14b, the preview point is 15.67 m in front of the driver.

**Figure 15:**
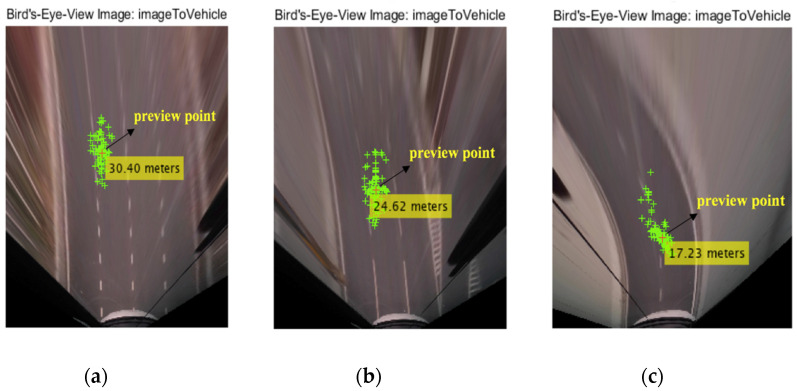
Bird’s-eye projection view of fixation points of subject 1 at a speed of 50 km/h on curves with radii of (**a**) 1500, (**b**) 800, and (**c**) 200 m.

Figure 15 shows the bird’s-eye projection view of the fixation points of subject 1 at a speed of 50 km/h on curves with radii of 1500, 800, and 200 m. At the same speed, as the radius of curvature decreases, the preview distance is correspondingly shorter.

### 5.6. Preview Time Model

Through the coordinate transformation, we obtained the longitudinal position $y_{g0}$ of the preview point of the eight subjects in the world coordinate system under different driving conditions—that is, the longitudinal (preview) distance from the driver to the preview point. The preview time of the driver is the duration for which the line of sight of the driver stays on the preview point. The estimation of the probability density of the preview time of the driver serves as an effective basis to describe the visual behavior of the driver, which can be directly applied for the automated lateral control of an intelligent vehicle. Therefore, we need to calculate the preview time according to the preview distance and establish the probability distribution model of the preview time as the driver preview model. As discussed in the previous section, the preview distance $y_{g0}$ can be calculated as

(27) $$y_{g0}=\frac{h_{21}^{\prime }x_{0}^{\prime }+h_{22}^{\prime }y_{0}^{\prime }+h_{24}^{\prime }}{h_{31}^{\prime }x_{0}^{\prime }+h_{32}^{\prime }y_{0}^{\prime }+h_{34}^{\prime }}$$

The fixed position camera is installed parallel to the vehicle axis, and there is no rotation in the yaw direction during coordinate projection—that is, the parameters $h_{21}^{\prime }$ and $h_{31}^{\prime }$ in Equation (27) are both 0 and the equation can be rewritten as

(28) $$y_{g0}=\frac{h_{22}^{\prime }}{h_{32}^{\prime }}-\frac{h_{32}^{\prime }h_{24}^{\prime }-h_{22}^{\prime }}{{\left(h_{32}^{\prime }\right)}^{2}y_{0}^{\prime }+h_{32}^{\prime }h_{34}^{\prime }}$$

where $y_{g0}$ is the ordinate of the preview point in the world coordinate system and $y_{0}^{\prime }$ is the vertical coordinate of the preview point in the image coordinate system. The preview time t of the driver can be expressed as

(29) $$t=\frac{h_{22}^{\prime }}{vh_{32}^{\prime }}-\frac{h_{32}^{\prime }h_{24}^{\prime }-h_{22}^{\prime }}{v[{\left(h_{32}^{\prime }\right)}^{2}y_{0}^{\prime }+h_{32}^{\prime }h_{34}^{\prime }]}$$

**Figure 16:**
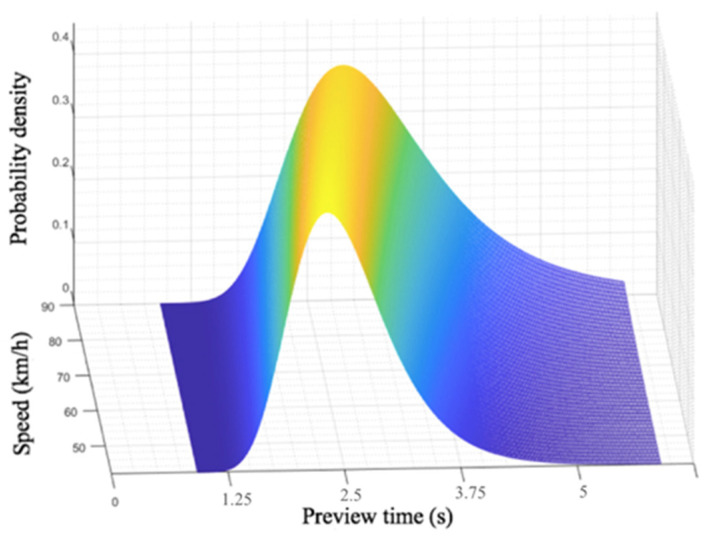
Probability density map of preview time on straight lane.

**Figure 17:**
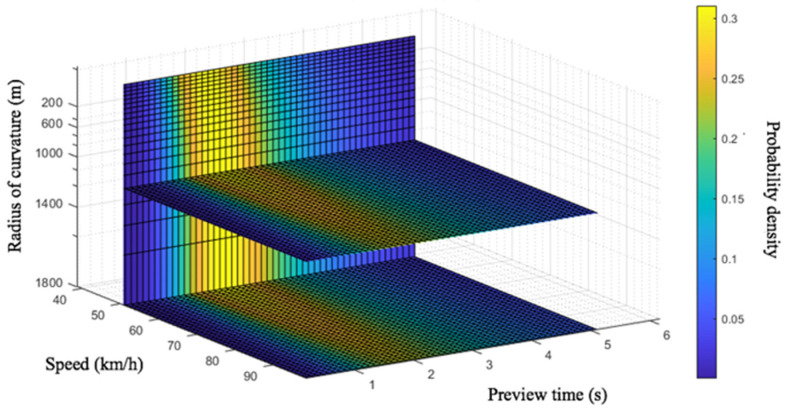
Probability density map of preview time on curves.

Combining Equation (29) for the preview time with Equations (19) and (22) for the distribution function of the fixation points for a straight lane and curves, respectively, the probability distribution of the preview time under different speeds and different conditions can be calculated, and a driver preview model suitable for all driving conditions and driving speeds can be obtained. Figure 16 and Figure 17 show the probability density map of the preview time on a straight lane and curves, respectively, at different driving speeds. It should be noted that the preview models in Figure 16 and Figure 17 are applicable to straight roads with a driving speed of 50 to 90 km/h and curves with a curvature range of 200 to 1800 m. That is, driving at medium and high speeds under relatively common straight and curved conditions can be applied to many common traffic scenarios, such as urban expressways and highways. For driving scenarios with higher speeds and with curves with smaller radii of curvature, such as highways and mountain roads, a specific experimental analysis should be carried out in subsequent studies.

It can be seen from Figure 16 that, at different speeds, the driver’s preview time is distributed in a fixed area, and the preview time increases with the vehicle speed. At the speed of 50 km/s, the peak of the preview time appears at 2.21 s. Likewise, the peak of preview time appears at 2.48 s at 70 km/h, and 2.89 s at 90 km/h. However, it is noteworthy that even the weights of these peak points are not large in absolute value—that is, the driver does not rely on an utmost accurate preview point in the process of visual recognition of the road. Interval preview is an important feature of the driver’s visual perception, and as the vehicle speed increases, the weight of the peak decreases. As showing in Figure 17, when driving on curves, both the vehicle speed and the road curvature radius have an impact on the driver’s preview time distribution. Their impacts are reflected in different aspects. The vehicle speed affects the dispersion degree of the preview time’s weight distribution. The higher the vehicle speed is, the lower the weight corresponding to the peak value of the probability density is. The radius of curvature affects the length of the preview time. The average preview time will decrease as the radius of road curvature decreases.

According to the distribution of the preview time under different driving conditions, the distribution weight of each moment can be determined. It provides a reference for the visual perception information of the actual driver while driving which can be used for constructing a control model to achieve automated path tracking under different driving speeds and curvature radii. We will incorporate it into the design of humanized driver control models in future work.

### 5.7. Model Validation

Figure 16 and Figure 17 are preview models fitted with specific parameters. The speed parameters used in the fitting process of the model are 50, 70, and 90 km/h; the radii of curvature are 200, 800, and 1200 m. Their applicability needs to be verified with more driving conditions. In order to verify the preview model of the straight lane, we selected 10 contrast speed values in the speed range of 60 km/h and 80 km/h, and calculated the preview time by Formulas (19), (22), and (29). Then the probability density peak value $u_{b}$ of analytical calculation was compared with the model fitting peak value $u_{f}$. As to the verification of the preview model of curve driving, we conducted a unilateral limit analysis on the driving speed and radius of curvature in the sampled data. The deviation calculation is the same as that of the above straight lane condition. Table 3 has given the comparison results of peak probability density between model fitting and analytical calculation under the straight and curve driving conditions.

We used the coefficient of determination $r^{2}$ to calculate the deviation between model fitting results and analytical calculation results [36]. The closer the $r^{2}$ is to 1, the smaller the deviation is. The calculation formula of $r^{2}$ is as follows:

(30) $$r^{2}=1-\frac{SS_{res}}{SS_{tot}}$$

where $SS_{res}$ is the total sum of squares of model probability density, and $SS_{tot}\;$ is the sum of squares of residuals. $SS_{res}$ and $SS_{tot}$ are calculated as follows:

(31) $$SS_{res}=\sum_{i=1}^{10}{\left(u_{bi}-u_{fi}\right)}^{2}$$

(32) $$SS_{tot}=\sum_{i=1}^{10}{\left(u_{bi}-{\bar{u}}_{bi}\right)}^{2}$$

where $u_{bi}$ represents the probability density peak value of the analytical calculation of sample *i* (i≤10), $u_{fi}$ represents the model fitting peak value of sample *i*, ${\bar{u}}_{bi}$ is the average value of the peak probability density of all samples.

The above formulas are used to calculate the judgment coefficients of the preview fitting model results and the actual analytical calculated results under two driving conditions of straight lane and curve, respectively. Finally, $r_{1}^{2}=0.965$ was calculated for straight lane conditions and $r_{2}^{2}=0.932$ for curve conditions, which illustrates the effectiveness of the model.

## 6. Conclusions

In the actual driving process, the driver will visually perceive the area in front of the lane and decide on the expected driving trajectory. We can utilize this visual behavior of the driver to construct a driver model and realize the automated motion control of driverless cars. In this study, we collected visual data of drivers through actual vehicle experiments and analyzed the visual perceptive characteristics of drivers. Our study mainly focuses on the following aspects:

(1) We designed actual vehicle experimental conditions with different driving speeds and curvature radii and collected and preprocessed the visual data of real drivers. The distribution characteristics of the fixation points of drivers under different driving conditions were statistically analyzed. The analysis and verification of the statistical distribution chart revealed that the fixation points of the driver have a normal distribution. Subsequently, the parameters of the normal distribution of the fixation points of each driver under different driving conditions were fitted to determine the preview point.
(2) A driver preview model was established based on the fitting of the driver’s perception data. In this way, a probability density map of the preview time can be obtained, which was determined by the road curvature and driving speed. The preview model was verified through the deviation analysis between the model fitted results and analytical calculation results. Under driving conditions of straight lane and curved roads, the correlation coefficients were 0.965 and 0.932, respectively, which shows a satisfactory performance.

This study can be extended in several ways. First, more driving scenarios (such as motorways), more road conditions (such as slopes and adhesion), and more driving maneuvers (such as lane changing and overtaking) can be conducted in experimental designs in follow-up studies so that the preview model based on the driver’s visual characteristics can be better adapted to more driving environments. Second, the driver preview model could be further improved by combining it with the physiological characteristics of drivers to realize a more humanized design.

## Figures and Tables

**Table 1:** Experimental conditions.

Route	Data Collection Time (s)		
	50 km/h	70 km/h	**90 km/h**
Straight lane	≥30	≥25	≥20
Curve, R = 1500 m	≥30	≥25	≥20
Curve, R = 800 m	≥30	≥25	≥20
Curve, R = 200 m	≥20	≥15	≥10

**Table 2:** Results of Jarque–Bera test for subjects 1–8 on straight lane.

Subject	50 km/h		70 km/h		90 km/h	
	JB Statistic	h	JB Statistic	h	JB Statistic	h
1	2.10	0	5.58	0	4.48	0
2	3.59	0	2.12	0	2.53	0
3	5.42	0	5.31	0	4.00	0
4	4.14	0	4.64	0	3.65	0
5	2.44	0	2.89	0	3.18	0
6	2.29	0	4.81	0	5.23	0
7	5.60	0	5.15	0	2.44	0
8	7.12	1	3.39	0	3.97	0

**Table 3:** Model validation.

Straight Lane					Curve					
*v* (km/h)	Preview Time (s)	*u_b_*	*u_f_*	$r_{1}^{2}$	*v* (km/h)	*R* (m)	Preview Time (s)	*u_b_*	*u_f_*	$r_{2}^{2}\;$
60	2.32	0.3922	0.3953	0.965	70	600	1.78	0.3298	0.3352	0.932
62	2.38	0.3893	0.3915		70	640	1.89	0.3312	0.3353	
64	2.41	0.3904	0.3890		70	680	2.05	0.3321	0.3366	
66	2.44	0.3855	0.3843		70	720	2.14	0.3291	0.3347	
68	2.46	0.3830	0.3842		70	760	2.24	0.3322	0.3361	
70	2.48	0.3825	0.3825		70	800	2.32	0.3369	0.3369	
72	2.54	0.3763	0.3788		72	800	2.33	0.3402	0.3421	
74	2.62	0.3756	0.3759		74	800	2.35	0.3564	0.3598	
76	2.69	0.3662	0.3694		76	800	2.37	0.3678	0.3664	
78	2.71	0.3623	0.3653		78	800	2.41	0.3802	0.3792	
80	2.77	0.3581	0.3612		80	800	2.43	0.3867	0.3852	
